# Fever-and-Ague

**Published:** 1879-08

**Authors:** 


### FEVER AND AGUE.

Dumb ague, malarial fever, bilious fever, etc., etc., are the same thing in fact, proceeding from the same cause and requiring the same treatment. The cause is "miasma," which means a rising from, as it is supposed to come up from the surface of the earth and impregnate the atmosphere, which, being breathed into the lungs, is taken into the circulation, and poisons the blood, causing it to be thick, sluggish, black and impure. In this condition the blood flows at first slowly, and at length scarcely moves at all at the extremities; and as the blood begins to die the instant it ceases to move, the limbs grow cold, the veins are distended, the fire of life goes out and the man dies—of congestive fever. Fever and ague is not generally considered to be a dangerous disease; but show me a man who has long suffered from the disease without proper treatment and I will show you a constitution racked and ruined.

The best preventive against fever is to keep indoors until an hour after sunrise, and then do not go out after sunset. A little fire should be kept burning in the grate or stove every morning and evening, from April to November.

*Treatment.*—Here again the treatment must be directed to the liver and spleen, which must be excited to action in order to relieve themselves of the thick blood with which they are gorged, preventing free circulation through the system. Both quinine and arsenic are injurious in this stage of the disease and should not be used. They will afford temporary relief, but they fix and fasten the disease in the system.

In "Dr. Hall's Family Doctor" book I have an exhaustive article on these diseases; and on page 114 I have given the recipe for pills, which I have used successfully in all of the many cases treated during twenty years of practice in the sickly portions of the South and Southwest. These pills have been somewhat improved, and another box of tonic pills, to be taken after the chills are cured, has been added, and the two boxes, in one package, are sold by druggists as "Dr. Hall's Complete Cure for Fever and Ague," at 50 cents; and although I have not the least interest in their sale, I must say that I believe them to be the most speedy and the safest remedy that can be found for the radical cure of fever and ague, and all malarial fevers. They do not contain quinine or arsenic.

RHEUMATISM.—While residing in the Southwest, I discovered a fact that seems to have been overlooked by other medical writers; that is that rheumatism is very frequently caused by malarial poisoning, and that the same treatment employed to cure fever and ague will often cure rheumatism. I would recommend this treatment in all cases of rheumatism, since it can do no harm, and the chances are that it will do positive good. The best time to take medicines, to counteract blood poisoning, is at night, the last thing before going to bed. The body and mind are then quiet, and the action of the medicine is more thorough.

Of all the ordinary diseases which afflict mankind, particularly in this country

